# Clinical Characteristics and Survival Outcomes in Neuroblastoma With Bone Metastasis Based on SEER Database Analysis

**DOI:** 10.3389/fonc.2021.677023

**Published:** 2021-06-01

**Authors:** Bin He, Jianshui Mao, Leyi Huang

**Affiliations:** ^1^ Department of Orthopedic Surgery, The Fourth Affiliated Hospital, Zhejiang University School of Medicine, Yiwu, China; ^2^ Department of Orthopedic Surgery, The Second Affiliated Hospital, Zhejiang University School of Medicine, Hangzhou, China; ^3^ Orthopedics Research Institute of Zhejiang University, Hangzhou, China; ^4^ Key Laboratory of Motor System Disease Research and Precision Therapy of Zhejiang Province, Hangzhou, China

**Keywords:** neuroblastoma, bone metastasis, clinical features, survival, risk factors

## Abstract

**Purpose:**

Clinical features and survival analysis of neuroblastoma (NB) are well explored. However, clinical research of NB patients with bone metastasis is rarely reported. Thus, the current study was performed to analyze the clinical features, survival outcome, and risk factors in those patients.

**Materials and Methods:**

We reviewed the Surveillance, Epidemiology, and End Results (SEER) database to select cases diagnosed with NB with bone metastasis from 2010 to 2016. Overall survival (OS) and cancer-specific survival (CSS) were analyzed through univariate Cox regression analysis. Subsequently, we performed multivariate analysis to determine independent predictors of survival. The Kaplan–Meier method was applied to intuitively show differences in prognostic value between independent risk factors.

**Results:**

We finally identified 393 NB patients with bone metastasis who were selected for survival analysis. Nearly half of the patients (47.3%) were aged >3 years. The adrenal gland was the primary tumor site, accounting for approximately two thirds of cases (66.2%). The 5-year OS and CSS rates of all patients were 62.1% and 64.1%, respectively. The univariate analysis indicated that age, lung metastasis, and tumor size were significantly associated with OS and CSS. Based on the multivariable analysis, age at 2 and 3 years, lung metastasis, and tumor size >10 cm remained significant negative predictors of OS and CSS.

**Conclusion:**

For NB patients with bone metastasis, three independent prognostic risk factors (age, lung metastasis, and tumor size) are helpful to clinicians for predicting prognosis and guiding treatment. Reasonable treatment modalities for these patients should be further investigated to prolong survival.

## Introduction

Neuroblastoma (NB) is one of the most common malignant solid tumors that occur in infants and young children, accounting for 15% of childhood tumor-related deaths (1). The adrenal gland is the most common primary site for NB, while bone is the most common site of distant metastasis of NB (2). Although patients with NB are linked to a good overall prognosis, those with metastasis usually have a poor survival outcome even after radical therapy (2, 3). Approximately 50% of patients with NB had distant metastasis at the time of diagnosis (4). The 5-year event-free survival rate of patients with high-risk NB is <50% (5). Treatment of NB currently includes surgical resection, chemotherapy, radiotherapy, autologous stem-cell transplantation, and immunotherapy (6). However, there is currently a lack of well-established treatment strategies for patients with metastatic NB. Additionally, the role of surgical resection of the primary tumor remains controversial.

At present, there are few large-scale clinical studies on NB with bone metastasis. This retrospective study based on the Surveillance, Epidemiology, and End Results (SEER) database, was conducted to investigate clinical characteristics and survival predictors among NB patients with bone metastasis. The aim of this study was to identify factors that could assist in the accurate prediction of survival for such patients.

## Materials And Methods

### Study Population

Data of all patients diagnosed with NB with bone metastasis from 2010 to 2016 were retrieved from the SEER database (https://seer.cancer.gov/). The database collects information on tumors from 20 geographic areas and is largely representative of the US population. This database is freely available and does not include patient identification information. This retrospective study was approved by the ethics committee of the Fourth Affiliated Hospital, Zhejiang University School of Medicine.

Based on the International Classification of Diseases for Oncology, Third Edition (ICD-O-3), we searched the case-listing section to select patients diagnosed with primary NB (ICD-O-3 histologic code: 9490, ganglioneuroblastoma; 9500, NB, not otherwise specified; 9504, spongioneuroblastoma; 9522, olfactory NB). According to the tumor stage, we only selected cases of NB with bone metastasis for analysis. NB and bone metastasis were synchronous. However, information on whether bone metastasis was the first metastatic site was not available. Patients without pathological diagnosis and those who did not receive chemotherapy were excluded. We extracted clinicopathological information from the cancer database, including race, continuous age, sex, tumor site, tumor grade, tumor size, distant metastasis, surgery, radiotherapy, chemotherapy, cause of death, duration of survival (months), and vital status. The main end points, namely overall survival (OS) and cancer-specific survival (CSS), were calculated as the time from diagnosis until death due to any reason and due to this malignant tumor, respectively (7, 8).

### Statistical Analyses

We used the SPSS version 21.0 software (IBM Corporation, Armonk, NY, USA) to perform all statistical analyses and draw the graphs. The Kaplan–Meier method was applied to intuitively show differences in prognostic value between independent risk factors. Univariate Cox regression analysis was performed using the following data: race, age, sex, tumor site, tumor grade, tumor size, surgery, radiotherapy, and organ metastasis. Significant prognostic factors identified in the univariate analysis were incorporated into a multivariate Cox regression analysis. We also presented the corresponding hazard ratio and 95% confidence interval to indicate the impact of each variable on survival. A two-sided P-value <0.05 denoted statistical significance.

## Results

### Clinical Features

**Table 1** summarizes the detailed clinical features of all patients. A total of 393 NB patients with bone metastasis were identified (217 males [55.2%] and 176 females [44.8%]). More than two thirds of the patients were White. Of note, 66 (16.8%), 69 (17.6%), 72 (18.3%), and 186 (47.3%) patients were aged <1, 1, 2, and >3 years, respectively. The adrenal gland was the primary tumor site, accounting for approximately two-thirds (66.2%) of cases. Histologically, more than half (53.2%) of the patients were diagnosed with Grade III. More than one third (43.0%) of them had a tumor size of 5 to 10 cm. The majority of patients (77.9%) underwent surgery, and more than half (53.9%) received radiotherapy. There were 43 (10.9%), 78 (19.8%), and 44 (11.2%) NB patients with brain, liver, and lung metastasis, respectively. At the time of data collection, 104 (26.5%) patients had expired. The 5-year OS and CSS rates of this population were 62.1% and 64.1%, respectively.

**Table 1:** Baseline characteristics of 393 NB patients with bone metastasis at diagnosis.

Variable	Value
**Race**	
Black	66(16.8%)
White	285(72.5%)
Others	42(10.7%)
**Age (years)**	
<1	66(16.8%)
1	69(17.6%)
2	72(18.3%)
≥3	186(47.3%)
**Sex**	
Female	176(44.8%)
Male	217(55.2%)
**Tumor site**	
Adrenal gland	260(66.2%)
Soft tissue	63(16.0%)
Retroperitoneum	45(11.5%)
Others	25(6.4%)
**Tumor grade**	
Grade I	6 (1.5%)
Grade II	2 (0.5%)
Grade III	209 (53.2%)
Grade IV	28 (7.1%)
Unknown	148 (37.7%)
**Tumor size**	
<5 cm	57 (14.5%)
5–10 cm	169 (43.0%)
>10 cm	92 (23.4%)
Unknown	75 (19.1%)
**Surgery**	
Yes	306 (77.9%)
No	87 (22.1%)
**Radiotherapy**	
Yes	212 (53.9%)
No	181 (46.1%)
**Brain metastasis**	
Yes	43 (10.9%)
No	350 (89.1%)
**Liver metastasis**	
Yes	78 (19.8%)
No	315 (80.2%)
**Lung metastasis**	
Yes	44 (11.2%)
No	349 (88.8%)

NB, neuroblastoma; OS, overall survival; CSS, cancer-specific survival.

### Univariate Cox Regression Analysis

**Figure 1:**
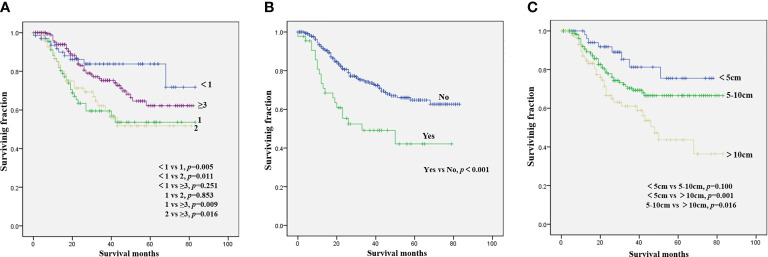
Kaplan–Meier method for the estimation of OS in NB patients with bone metastasis at diagnosis stratified by age **(A)**, lung metastasis **(B)**, and tumor size **(C)**. NB, neuroblastoma; OS, overall survival.

**Figure 2:**
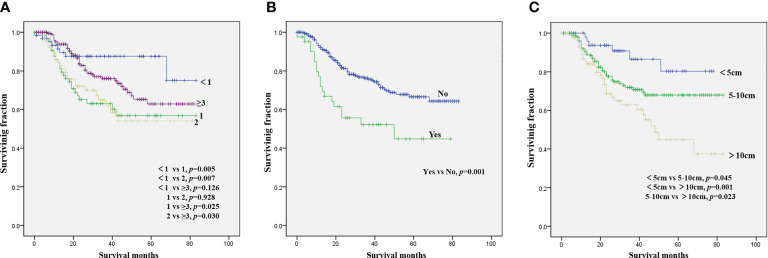
Kaplan–Meier method for the estimation of CSS in NB patients with bone metastasis at diagnosis stratified by age **(A)**, lung metastasis **(B)**, and tumor size **(C)**. NB, neuroblastoma; CSS, cancer-specific survival.

The results of the univariate analysis of NB patients with bone metastasis are presented in **Table 2**. Race, sex, tumor site, and tumor grade did not affect prognosis. Patients aged <1 year were associated with the best OS (**Figure 1A**) and CSS (**Figure 2A**). Moreover, tumor size >10 cm was significantly correlated with worse OS (**Figure 1C**) and CSS (**Figure 2C**). Surgery and radiotherapy did not confer an advantage to the survival of this population. Compared with patients without lung metastasis, those with lung metastasis had a worse prognosis (**Figures 1B** and **2B**). Brain or liver metastasis was not a risk factor for survival.

**Table 2:** Univariate Cox analysis of variables in NB patients with bone metastasis at diagnosis.

Variable	OS		CSS	
	HR (95% CI)	*p*	HR (95% CI)	*p*
**Race**				
Black	1		1	
White	1.228 (0.715–2.109)	0.456	1.498 (0.812–2.762)	0.196
Others	1.748 (0.852–3.586)	0.127	2.105 (0.959–4.619)	0.063
**Age (years)**				
<1	1		1	
1	2.746 (1.328–5.676)	0.006	3.074 (1.380-6.846)	0.006
2	2.618 (1.256–5.456)	0.01	3.008 (1.344–6.731)	0.007
≥3	1.467 (0.736–2.923)	0.277	1.753 (0.822–3.741)	0.146
**Sex**				
Female	1		1	
Male	1.167 (0.790–1.724)	0.439	1.114 (0.743–1.669)	0.601
**Tumor site**				
Adrenal gland	1		1	
Soft tissue	0.585 (0.311–1.103)	0.098	0.633 (0.334–1.197)	0.16
Retroperitoneum	1.123 (0.622–2.025)	0.701	1.130 (0.611–2.090)	0.696
Others	1.093 (0.475–2.517)	0.834	1.162 (0.503–2.686)	0.725
**Tumor grade**				
Grade III	1		1	
Grade IV	1.678 (0.917–3.072)	0.093	1.798 (0.976–3.310)	0.06
**Tumor size**				
<5 cm	1		1	
5–10 cm	1.873 (0.879–3.990)	0.104	2.337 (0.991–5.514)	0.052
>10 cm	3.230 (1.498–6.966)	0.003	3.964 (1.660–9.464)	0.002
**Surgery**				
Yes	1		1	
No	1.001 (0.625–1.602)	0.998	0.981 (0.599–1.606)	0.939
**Radiotherapy**				
Yes	1		1	
No	1.148 (0.781–1.688)	0.483	1.136 (0.760–1.698)	0.533
**Brain metastasis**				
No	1		1	
Yes	1.312 (0.747–2.306)	0.344	1.413 (0.801–2.492)	0.232
**Liver metastasis**				
No	1		1	
Yes	1.315 (0.838–2.063)	0.233	1.306 (0.817–2.088)	0.264
**Lung metastasis**				
No	1		1	
Yes	2.342 (1.450–3.783)	0.001	2.352 (1.423–3.888)	0.001

NB, neuroblastoma; OS, overall survival; CSS, cancer-specific survival).

### Multivariate Cox Regression Analysis

**Table 3** presents the results of the multivariate analysis of NB patients with bone metastasis. Patient age of 1 and 2 years was a beneficial factor for OS and CSS. In contrast, tumor size >10 cm was significantly correlated with worse rates of OS and CSS. OS and CSS were significantly improved in patients without lung metastasis versus those with lung metastasis.

**Table 3:** Multivariate Cox analysis of variables in NB patients with bone metastasis at diagnosis.

Variable	OS		CSS	
	HR (95% CI)	*p*	HR (95% CI)	*p*
**Age (years)**				
<1	1		1	
1	2.576 (1.235–5.372)	0.012	2.849 (1.269–6.397)	0.011
2	2.774 (1.317–5.841)	0.007	3.125 (1.384–7.054)	0.006
≥3	1.371 (0.678–2.770)	0.38	1.615 (0.748–3.489)	0.222
**Tumor size**				
<5 cm	1		1	
5–10 cm	1.797 (0.840–3.841)	0.131	2.231 (0.943–5.277)	0.068
>10 cm	2.872 (1.317–6.260)	0.008	3.466 (1.438–8.355)	0.006
**Lung metastasis**				
No	1		1	
Yes	2.390 (1.469–3.889)	<0.001	2.347 (1.412–3.902)	0.001

NB, neuroblastoma; OS, overall survival; CSS, cancer-specific survival.

## Discussion

To our knowledge, this retrospective study was the largest investigation of NB patients with bone metastasis and the first to perform a survival analysis of this population. The present study revealed the clinical features of NB patients with bone metastasis, which may be helpful to clinicians for understanding the epidemiological characteristics and diagnosis of such patients. This study foremost identified age at diagnosis, lung metastasis, and tumor size as independent predictors of OS and CSS. These factors may assist clinicians in predicting survival outcomes and personalizing treatment in this setting.

Compared with patients with low- and intermediate-risk NB (9), those who present with bone metastasis have unfavorable outcomes with a 5-year OS rate of 62.1% and a CSS rate of 64.1%. Consistent with the results of other studies (10, 11), sex was not associated with survival. In accordance with prior studies of NB, the present analysis validated the independent prognostic ability of age for NB patients with bone metastasis (12, 13). This study demonstrated that patients aged 1 or 2 years had a significantly worse prognosis than those aged <1 year or aged ≥3 years. Although the prognosis of patients with cancer aged >3 years was worse than that of patients aged <1 year, there was no statistically significant difference in prognosis between these two groups. Morgenstern et al. (14) reported that aged ≥18 months at diagnosis was associated with worse outcome of neuroblastoma. Patients aged 1 or 2 years with the worst prognosis may be a characteristic of the special group of NB patients with bone metastasis. Further studies should be performed to confirm the effect of age on the survival of NB patients with bone metastasis. Consistent with findings on overall NB (11, 15), race did not significantly influence survival in this population. Although some studies have suggested that the tumor site was related to the prognosis of NB (16, 17), we did not observe an association between tumor site and survival time in this analysis. Tumor size was identified as an independent predictor of OS and CSS, corroborating evidence reported by recent studies (17, 18). However, Zhou et al. (19) found that tumor size did not exert a significant prognostic effect on the rate of event-free survival. Among NB patients with bone metastasis, those with additional lung metastasis were linked to significantly worse survival, suggesting that multidisciplinary treatment of such patients is essential.

Although more intensive treatment is necessary for patients with metastatic NB (20), optimal treatment modalities for NB patients with bone metastasis have not been determined. Since the vast majority of NB patients with bone metastasis receive chemotherapy, this study only included patients who had undergone this type of treatment. Vollmer et al. (21) identified the value of radical surgery for patients with metastatic NB; radical surgery was an independent favorable factor for improved survival. However, some researchers did not observe a survival benefit of surgical resection in patients with high-risk NB (22, 23). According to these previous studies, there was no significant difference in survival between NB patients with bone metastasis who underwent surgery at the primary site and those who did not. Typically, radiotherapy is used as a palliative treatment for patients with metastatic NB to relieve local symptoms and control local recurrence (24, 25). Hu et al. (26) reported that radiotherapy prolongs the survival time of NB patients with brain metastasis. In the present analysis, we confirmed that radiotherapy was not associated with survival in such patients. Additionally, toxicity of radiotherapy in patients with NB was recently noted (27, 28). Therefore, improvements in the treatment of these patients are urgently required.

This study had several limitations that must be considered. Firstly, this retrospective study may be characterized by selection bias during the survival analysis. Secondly, data on some other treatment modalities, including immunotherapy, retinoid therapy, and stem-cell transplantation, were unavailable in the SEER database. It has been shown that MYCN amplification plays an important role in the prognosis of NB (14); in combination with bone metastasis, this alteration determines an ultra-high-risk group of patients with NB (29). However, information on the MYCN status was not available in the SEER database. Future research studies should examine the role of MYCN amplification in this setting. Nevertheless, there are strengths of this study that should be noted. The SEER database can be used to conduct clinical studies of rare types of cancer. In addition, this database provides the possibility of multi-center research, and the results are more extrapolatable to wider populations.

## Conclusions

This study confirmed three valuable prognostic factors in NB patients with bone metastasis, namely age, lung metastasis, and tumor size. Surgery and radiotherapy were not identified as valuable factors in prolonging the survival of NB patients with bone metastasis. Thus, the effective treatment of these patients remains a challenge for clinicians.

## Data Availability Statement

The original contributions presented in the study are included in the article/supplementary material. Further inquiries can be directed to the corresponding author.

## Author Contributions

BH conceived of and designed the study. JM and LH collected the data. BH, JM, and LH performed the statistical analysis. BH wrote the manuscript. BH and JM revised it. All authors contributed to the article and approved the submitted version.

## Conflict of Interest

The authors declare that the research was conducted in the absence of any commercial or financial relationships that could be construed as a potential conflict of interest.
